# National COVID-19 preparedness and response plans: a global review from the perspective of services for maternal, newborn, child and adolescent health and older people

**DOI:** 10.1136/bmjgh-2023-013711

**Published:** 2024-03-04

**Authors:** Alexandra Czerniewska, Alyssa Sharkey, Anayda Portela, Sarah Drapkin, Saqif Mustafa

**Affiliations:** 1 Health Equity and Human Rights LLC, Princeton, New Jersey, USA; 2 School of Public and International Affairs, Princeton University, Princeton, New Jersey, USA; 3 Department of Maternal, Newborn, Child and Adolescent Health, World Health Organization, Geneva, Switzerland; 4 Princeton University, Princeton, New Jersey, USA; 5 Integrated Health Services, World Health Organization, Geneve, Switzerland

**Keywords:** COVID-19, Maternal health, Child health, Review

## Abstract

**Introduction:**

Infectious disease outbreaks have historically led to widespread disruptions in routine essential health services. Disruptions due to COVID-19 responses led to excess deaths, including among women and children. This review builds on earlier reviews of essential health services in national COVID-19 response and preparedness plans, focusing specifically on maternal, newborn, child, adolescent and ageing health (MNCAAH) in the context of renewed global emphasis on monitoring, recovering and strengthening these services.

**Methods:**

Using Google searches, we identified publicly available COVID-19 response and preparedness plans authored by a national government body or Public Health Institute from any country, territory and/or area, published between January 2020 and December 2022. We assessed whether each plan considered maintenance of MNCAAH services with related activities, costing or monitoring plans, and whether these considerations were integrated into the national incident management system for COVID-19.

**Results:**

We identified plans from 110 countries, representing 56% of our sample, in 10 languages. Most plans came from low-income and middle-income countries. Three quarters of dated documents were published between February and April 2020. 22% of plans referenced the impact of COVID-19 on MNCAAH, but only 13% included a planned activity for monitoring or mitigating this impact and less than 5% included relevant indicators, costing or integration of services in the incident management system.

**Conclusion:**

We propose that unless content specifically related to the services and needs of these populations is integrated, these services will suffer in a future disruptive event. The COVID-19 response demonstrated the need for an interdisciplinary response to address the unforeseen impacts that arose, yet plans continue to have a narrow focus and a generic approach which may be limiting.

**WHAT IS ALREADY KNOWN ON THIS TOPIC**

- There were excess deaths among women and children during the COVID-19 pandemic from preventable causes because of disruptions to essential health services. Earlier reviews suggested that national plans and policies published in 2020 had limitations in their consideration of essential health services alongside immediate COVID-19 response.

**WHAT THIS STUDY ADDS**

- This is the first study to examine country considerations of maternal, newborn, child and adolescent health services, and health services for older people, within national COVID-19 preparedness and response plans. Few countries incorporated considerations for these health services into their COVID-19 plans, and even fewer described specific activities, indicators or resource allocations to mitigate potential service disruptions during the COVID-19 pandemic.

**HOW THIS STUDY MIGHT AFFECT RESEARCH, PRACTICE OR POLICY**

- Commitments and investments in primary healthcare are critical to ensure continuation of lifesaving and essential services during a large-scale outbreak or public health event and to avoid excess and preventable mortality and morbidity. Maintenance of essential health services for specific population health services and needs requires explicit mention in policies and plans with clear actions such as resourcing and monitoring for avoiding service disruptions.

## Introduction

### Disruption to essential health services

Infectious disease outbreaks and other disruptive public health events such as natural disasters, war and conflict have historically led to widespread disruption in use of routine essential health services.^1–4^ Surveys and studies during the COVID-19 pandemic showed a decrease in the use of essential health services in most countries, especially in the early months of the pandemic^5–7^ but continuing over longer periods^8^: disruptions to sexual, reproductive, maternal, newborn, child and adolescent health services were reported by 33% of countries in the final quarter of 2021, down from 55% in the third quarter of 2020,^5^ and a continued reduction in immunisation coverage was observed over 2020 and 2021.^9^

In addition to the nearly 7 million deaths reported to WHO as of 24 May 2023, estimates suggest that there were another additional 15 million excess deaths (range 13.3–16.6 million) associated with the COVID-19 pandemic in 2020 and 2021 which were at least in part attributable to disruptions to essential health services,^10^ as well as substantial underreporting of COVID-19 deaths. Although there is a dearth of data to quantify the precise contribution of each cause,^11 12^ one modelling study estimated that there were 114 000 excess child and maternal deaths across 18 low-income and lower-middle-income countries between March 2020 and June 2021, based on observed average health service declines of 2.6% to 4.6% for maternal and child services.^13^ The United Nations Population Fund estimated that the pandemic disrupted contraceptive use for about 12 million women with a consequence of nearly 1.4 million unintended pregnancies during 2020 across 115 low-income and middle-income countries.^14^ Early in the pandemic it was estimated that for every excess death attributable to coronavirus infections acquired during routine visits to vaccination clinics, 84 child deaths (95% uncertainty interval: 14–267) could be prevented by sustaining routine childhood immunisation in Africa.^15^

Disruption to services during the COVID-19 pandemic reflected supply issues such as disruptions to supply chains, shortages of personal protective equipment, reduced staffing and diverting resources towards COVID-19 services, reduced access to services through restrictions to movement and transportation and reduced demand through inability to pay for services, or uncertainty about the safety of accessing care.^16 17^ Mitigating the effect on essential services during disruptive public health events is therefore especially challenging in settings with fewer health sector resources, greater health and financial needs in the population, and/or weaker health sector governance. Certain populations are also disproportionately affected by the disruption of routine health services. For example, older people may have ongoing needs for medication and care, including home-based visits and community care, women require time-sensitive care during pregnancy and childbirth, and newborns and young children are most susceptible to preventable and treatable diseases requiring urgent care such as sepsis, pneumonia, diarrhoea and malaria.^18^

### COVID-19 preparedness and response plans (CPRPs)

In response to the COVID-19 outbreak, countries developed national response plans. Many countries based their plans on global guidance from the WHO, which published the first strategic preparedness and response plan (SPRP) on 4 February 2020,^19^ with updates in April 2020^20^ and February 2021^21^ and accompanying operational plans.^22 23^ The early SPRPs set out guidance for country-level planning—specifically advising that countries consider eight thematic ‘pillars’ of: country-level coordination, planning and monitoring; risk communication and community engagement; surveillance, rapid-response teams and case investigation; points of entry; national laboratories; infection prevention and control (IPC); case management, and operations support and logistics. Alongside this, WHO published guidance on maintaining essential health services, with the intention that countries would include this in the COVID-19 planning process. The first of these in March 2020^24^ was short and high level, but it was updated with substantially more detail in June 2020^25^ to include key actions that countries should consider to ensure the continuity of essential health services, and especially advising adaptations, considerations and monitoring indicators for life course and disease programmes. This June 2020 document in particular provided countries with detailed processes to plan services for maternal, newborn, child, adolescent and ageing health (MNCAAH) in the context of COVID-19, and the SPRP update in February 2021^21^ included a ‘Pillar 9’ on strengthening essential health services and systems.

### Current data and gaps

Earlier reviews have suggested that national plans and policies published until the later months of 2020 had limitations in their consideration of essential health services alongside immediate COVID-19 response to stop the pandemic,^26 27^ their overall processes to prioritise scarce healthcare resources^28–30^ or their consideration of the needs of specific populations.^31 32^

This review builds on the earlier review of integration of essential health services in national CPRPs by Mustafa *et al*.^26^ Our review focuses specifically on the integration of MNCAAH in CPRPs. We aim to identify CPRPs published over almost 3 years of the pandemic in the context of renewed global emphasis on, and guidance for, monitoring and maintaining essential services and identify content specific to maintaining essential MNCAAH services or directed to the health needs of these populations.^33 34^

### Objectives of the review

1. To identify national CPRPs with content related to MNCAAH.
2. To extract and summarise content related to MNCAAH included in these plans and determine whether they consider:
  - The impact of COVID-19 on essential MNCAAH services.
  - Activities for the maintenance of safe, quality and routine essential MNCAAH services during the COVID-19 outbreak.
  - Resources/budget for maintaining MNCAAH services.
  - Integration of MNCAAH into the national incident management system (IMS).

## Methods

We updated a database of publicly available national COVID-19 planning documents compiled in 2020.^26^ We searched for new and updated plans across national government, Ministry of Health and Public Health Institute webpages and using search terms in Google. We designed search terms related to COVID-19, response planning and variations of the country name in English, Spanish, French and Portuguese, although plans in any language were eligible for inclusion (see online supplemental material). We further searched reference lists from other literature identified through our Google search, for example, reviews of national response efforts. We also contacted authors of a paper containing similar analyses to identify plans.^29^

10.1136/bmjgh-2023-013711.supp1 Supplementary data

Three reviewers (AC, SD, AS) screened documents for eligibility. We included country COVID-19 planning documents authored or co-authored by a national government, a national government ministry or a Public Health Institute in any WHO member country, territory, or area, published between January 2020 and December 2022 in any language and in any format (eg, PDF, webpage). Where more than one plan was identified for a country, for example, we included the most recent version only. We included plans that explicitly listed the government as an author or included a government logo. We excluded plans that only described activities for non-governmental organisations, plans related to actions for non-health sectors (eg, education, transport), plans related to general or non-COVID-19 health emergencies or outbreaks, plans covering only part of a country, retrospective reports of COVID-19 activities, summaries or presentations about a national plan, legal documents/decrees without associated action plans or documents that were primarily aimed at informing the public about their personal responsibilities (table 1).

**Table 1:** Inclusion and exclusion criteria

Criteria	Included	Excluded
Location	WHO member country, territory, or area	Plans from other countries, territories, or areas
Date	January 2020–December 2022	Outside this date range
Language	Any language	
Authorship	National government, national government ministry or national public health institute. With or without another organisational author	Plans without a national government author or co-author, judged by name or logo appearing on document
Format	Any format	
Type	Most recent national, prospective COVID-19 response plans for the health sector, including the government’s health response	Plans describing activities for non-health sectors (eg, education, transport) or non-government organisations only Older versions of updated plans Plans related to general or non-COVID-19 health emergencies or outbreaks Plans primarily aimed at informing the public about their personal responsibilities Sub-national plans Retrospective reports Summaries of, or presentations about, a plan Legal documents/decrees without associated plans

For each plan, AC, AS or SD extracted information on the name of the plan, organisational authors, date published and language, and data related to maintaining the provision and use of MNCAAH services, combining manual reading of each document with keyword searches targeting multiple aspects of MNCAAH services to ensure relevant sections were identified. Articles not in English were translated by authors or by using the online translation software Google Translate, with clarifications sought from fluent speakers where necessary. In a subsample of 20% of CRPRs, a second author (AC, AS or SD) independently extracted data to check for discrepancies.

### Data analysis

We recorded whether the documents included a mention of essential MNCAAH services, that is, the impact on services or the need to maintain them, however substantive, whether they considered how to ensure maintenance of safe, quality, essential MNCAAH services, integrated MNCAAH into the national IMS or included relevant costing or monitoring plans for MNCAAH activities or outcomes. We defined essential MNCAAH services as per WHO guidance^25 35^ (see respective annexes): maternal and newborn health, child health, immunisation, nutrition, sexual and reproductive health, gender-based violence (GBV), adolescent health, mental health and services providing healthcare—including residential care—for older people. Outside of the scope of this review were content for services related to childcare and child protection, strengthening of COVID-19 IPC or triage in health facilities, priority COVID-19 testing or vaccination for vulnerable populations, COVID-19 case management protocols or psychosocial care specific to COVID-19.

We analysed our findings by the World Bank 2023 income group classifications^36^ and by WHO regions.

### Patient and public involvement

No patients or members of the public were involved in the design, conduct or reporting of this research.

## Results

### Overview

We identified eligible plans from 110 countries, representing 56% of WHO member countries, territories and areas. The distribution of plans included by the World Bank income classification^36^ was: 23% low-income countries (n=25); 39% lower-middle-income countries (n=43); 23% upper-middle-income countries (n=25); 12% high-income countries (n=13) and 4% were countries not classified by the World Bank due to lack of available data (n=4). A higher proportion of plans were publicly available in lower-income regions—we identified a plan for 89% of low-income countries (n=25) and 81% of lower-middle income countries (n=43), versus 48% of upper-middle income countries (n=25) and 22% of high-income countries (n=13).

The distribution by WHO region was: 40% African Region (n=44); 17% Region of the Americas (n=19); 14% Eastern Mediterranean Region (n=15); 11% European Region (n=12); 6% South-East Asian Region (n=7) and 12% Western Pacific Region (n=13) (table 2).

**Table 2:** Number of countries, territories and areas included in this study, by World Bank income group classification^36^ and WHO region

	Total WHO member countries, territories and areas	CPRPs included (as a % of total included)
Income level by World Bank classification		
Low	28	25 (23%)
Lower-middle	53	43 (39%)
Upper-middle	52	25 (23%)
High	58	13 (12%)
Not classified	4	4 (4%)
Total	195	110 (100%)
WHO region		
African Region	47	44 (40%)
Region of the Americas	35	19 (17%)
Eastern Mediterranean Region	22	15 (14%)
European Region	53	12 (11%)
South-East Asian Region	11	7 (6%)
Western Pacific Region	27	13 (12%)
Total	195	110 (100%)

CPRPs, COVID-19 preparedness and response plans.

We found plans in 10 languages: English (n=60, 55%); French (n=21, 19%); Spanish (n=14, 13%); Portuguese (n=7, 6%); Arabic (n=3, 3%); Croatian (n=1, 1%); Danish (n=1, 1%); Indonesian (n=1, 1%); Nepali (n=1, 1%) and Romanian (n=1, 1%). National government authorities were the sole authors in 98 of the 110 included CPRPs (89%), for example, Ministries of Health, National Public Health Institutes, National Centres for Disease Control, Central Government offices or National Taskforce groups. The WHO was listed as co-author in 12 CPRPs (11%).

**Figure 1:**
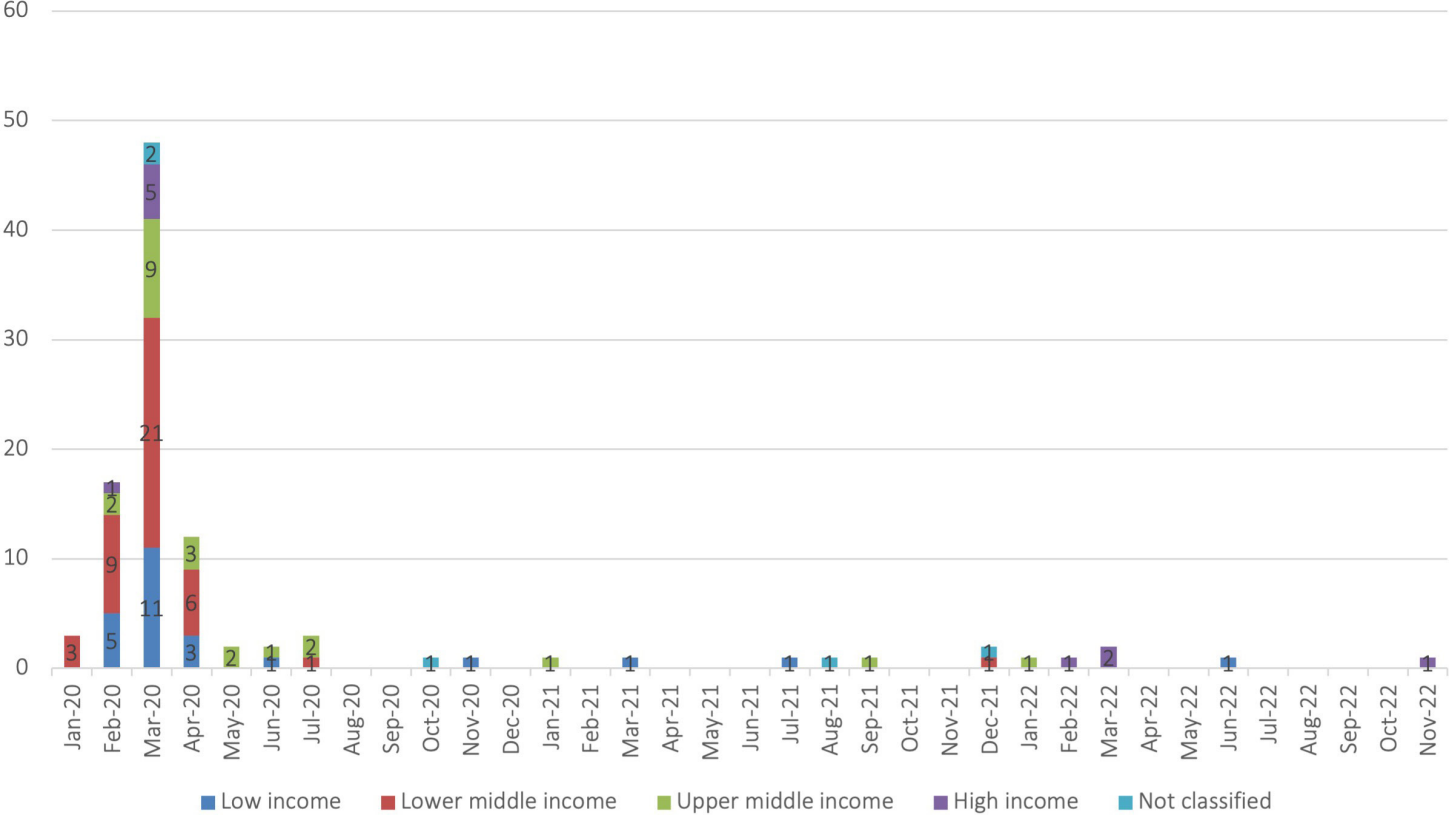
CPRPs by month authored and World Bank income group classification (n=102 dated plans). CPRPs, COVID-19 preparedness and response plans.

The plans were authored between January 2020 and November 2022 (figure 1), with three quarters of dated documents authored in the 3-month period from February and April 2020 (n=77, 75% of 102 dated documents). Eight plans did not provide a month of publication. Of these, one was dated 2021; one was dated 2022; four we estimated to be from February/March 2020; one we estimated to be from October 2020 and one we could not estimate. It was not possible to report the version number of the included plans, or whether they were updated from original plans, due to inconsistent reporting of this information.

### Essential services and MNCAAH content

Of the 110 included CPRPs, 40 (36%) included content related to maintaining essential health services, although only 31 (28%) of these CPRPs had a dedicated section/pillar for this. Only 24 (22%) considered of the impact of the COVID-19 outbreak on one or more MNCAAH service, and/or the need to protect or adapt these services (table 3). Child health services were the most frequently mentioned (n=20 CPRPs; 18%), especially in relation to continuing childhood immunisation programmes, followed by maternal/newborn services (n=18; 15%), services for older people (n=10; 9%), sexual and reproductive health services (n=10; 9%), GBV (n=3; 3%) and adolescent health services (n=2; 2%). No CPRP considered services for all MNCAA populations.

**Table 3:** Content related to MNCAAH services identified in CPRPs, by World Bank income group classification and WHO region

	CPRPs included	Considers the impact of COVID-19 on MNCAAH services*	Plans activities for maintenance of MNCAAH services†	Includes indicators for monitoring MNCAAH services/activities‡	Considers budget for maintaining MNCAAH services§	Integrates MNCAAH into the wider COVID-19 IMS¶
Income level by World Bank classification						
Low	25	9 (36%)	6 (24%)	2 (8%)	0 (0%)	2 (8%)
Lower-middle	43	6 (14%)	4 (9%)	2 (5%)	2 (5%)	0 (0%)
Upper-middle	25	6 (24%)	3 (12%)	0 (0%)	1 (4%)	0 (0%)
High	13	2 (15%)	1 (8%)	0 (0%)	0 (0%)	0 (0%)
Not classified	4	1 (25%)	0 (0%)	0 (0%)	0 (0%)	0 (0%)
Total	110	24 (22%)	14 (13%)	4 (4%)	3 (3%)	2 (2%)
WHO region						
African Region	44	9 (20%)	6 (14%)	2 (5%)	0 (0%)	2 (5%)
Region of the Americas	19	3 (16%)	2 (11%)	0 (0%)	0 (0%)	0 (0%)
Eastern Mediterranean Region	15	3 (20%)	2 (13%)	1 (7%)	1 (7%)	0 (0%)
European Region	12	0 (0%)	0 (0%)	0 (0%)	0 (0%)	0 (0%)
South-East Asian Region	7	5 (71%)	3 (43%)	1 (14%)	2 (29%)	0 (0%)
Western Pacific Region	13	4 (31%)	1 (8%)	0 (0%)	0 (0%)	0 (0%)
Total	110	24 (22%)	14 (13%)	4 (4%)	3 (3%)	2 (2%)

*Includes at least one mention of the impact of COVID-19 on at least one MNCAAH service. We accepted a simple statement about the need for an MNCAAH service or population to be protected.

†Includes at least one activity for the maintenance of at least one MNCAAH service during the COVID-19 outbreak. We accepted activities related to future planning for MNCAAH services.

‡Includes at least one cost of an activity relating to at least one MNCAAH service.

§Includes at least one relevant indicator for monitoring the functioning of an MNCAAH service, or any relevant activity output, outcome or impact.

¶Includes reference to how MNCAAH services will be considered by the Incident Management System (IMS), or report into it, for example a working group.

CPRPs, COVID-19 preparedness and response plans; MNCAAH, maternal, newborn, child, adolescent and ageing health.

The extent of consideration of these services varied, and many CPRPs only included a simple statement about the need for a service or population to be protected. In 14 of these 24 CPRPs (13% of total), a planned activity accompanied this consideration. In five CPRPs, the activities focused on strengthening community level or home-based care through strengthening existing facilities (Bangladesh), creating new temporary facilities (Zambia), reorganising towards more home-based care (Colombia, Ethiopia, Indonesia and Zambia) or investing in technology for virtual primary health service delivery (Sri Lanka). One CPRP included activities for protecting older people in residential care (Australia). Two CPRPs included activities relating to GBV (Pakistan and Uganda). Six CPRPs planned activities to assess of the needs for certain MNCAAH services (Central African Republic, Guyana, Malawi, Pakistan Somalia and South Africa). Only a small number of CPRPs included indicators for monitoring MNCAAH services or relevant activities (n=4; 4%), included costing of activities for MNCAAH services (n=3; 3%) or detailed the integration of MNCAAH into the wider IMS (n=2; 2%). No countries included all five of the population health areas, two countries—Malawi and Pakistan—included four, and four countries—Indonesia, Sri Lanka, Uganda and Zambia—included three, of which Indonesia had the most substantive MNCAAH content (Box 1).

Box 1 **Details from the three documents with the most substantive MNCAAH service planning,**

**Indonesia’s**

‘Pedoman Pencegahan Dan Pengendalian Coronavirus (COVID-19)’ [Coronavirus Disease (COVID-19) Prevention and Control Guidelines] from July 2020 included a section on essential health services with recommendations for service providers. Family health (including maternal, newborn and child health services), family planning and immunisation services were identified as essential health services to maintain. This plan included a comprehensive set of recommendations for reorganising primary healthcare services and retaining health workers during the COVID-19 outbreak and provided links to a Google Drive containing more detailed guidelines specific to a range of services including antenatal services through labour, postnatal care for the woman and newborn. Several indictors were recommended to monitor these services: First ANC visit to pregnant women; number of births at health facilities; number of infants under 1 year who received the third dose of diphtheria tetanus-pertussis (DPT3) immunisation or the first dose of measles immunisation; number of women who received (a) oral and (b) injectable contraception, and number of children aged 0–59 months who attended the health facility to receive treatment for malnutrition (wasting) and bilateral pitting oedema. Other MNCAAH services were not mentioned, for example, for adolescents and older people.

**Malawi’s**

‘National COVID-19 Preparedness and Response Strategy and Plan’ from July 2021 included four primary goals, including to ‘support sustainability of essential health services while containing the COVID-19 epidemic’. Detailed action plans with costing and monitoring and evaluation plans were assigned to ‘clusters’ (working groups) on ‘Health’ and ‘Protection & Social Support’ which reported into the IMS. Costed activities and indicators were included for assessing public and private Maternal/Under 5 health services care, advocating with health services to provide Sexual and Reproductive Health and Rights (SRHR) services to adolescent girls and young women and conducting SRHR and Gender-Based Violence (GBV) awareness sessions with adolescent girls and young women linking them services in health facilities. This plan included relevant indicators with targets, verifiable through the health management information system of: Outpatient service utilisation (visits per 1000 population); % of children under 1 year of age fully immunised and % births attended by skilled personnel. This plan did not consider health services for older people, or adolescent health services beyond including them in sexual and reproductive health services.

**Pakistan’s**

‘Preparedness and Response Plan COVID-19’ from April 2020 recognised, even relatively early in the pandemic, that COVID-19 had ‘the potential to reverse the reproductive health gains achieved so far and make existing vulnerabilities worse, limiting women’s access to lifesaving maternal health services as a result of movement restrictions, combined with the fear and household tensions’. Under Pillar 7 (Case Management) the plan included actions to continuously assess the burden on primary healthcare services and to ensure continuity of essential services including nutrition, reproductive health including child health and vaccination, and provision of essential medicines for child and maternal care. Sub actions included mapping vulnerable population including women and young girls, persons with disabilities, transgender community and youth/adolescents to the nearest health facility and establishing an incident reporting mechanism for addressing GBV incidents within communities. Actions were accompanied by indicators and supported by high-level budget lines.This plan did not specifically mention any health services for older people, although we assume they fall under the ‘vulnerable populations’ category’.

Analysing by income group, we observed the highest proportion of countries that considered the impact of COVID-19 on MNCAAH services and included at least one MNCAAH activity in low-income countries (36% considered impact; 24% included an activity), but we detected no linear pattern across the other income levels. Analysing by WHO regions, by far the highest proportions of plans that considered impact or included a relevant activity was observed in the South-East Asian Region (71% considered impact; 43% included an activity) although the number of plans in that region was small (7 plans included out of 11 countries in the WHO region). Conversely, none of the 12 plans included from the European Region considered MNCAAH services (table 3).

**Figure 2:**
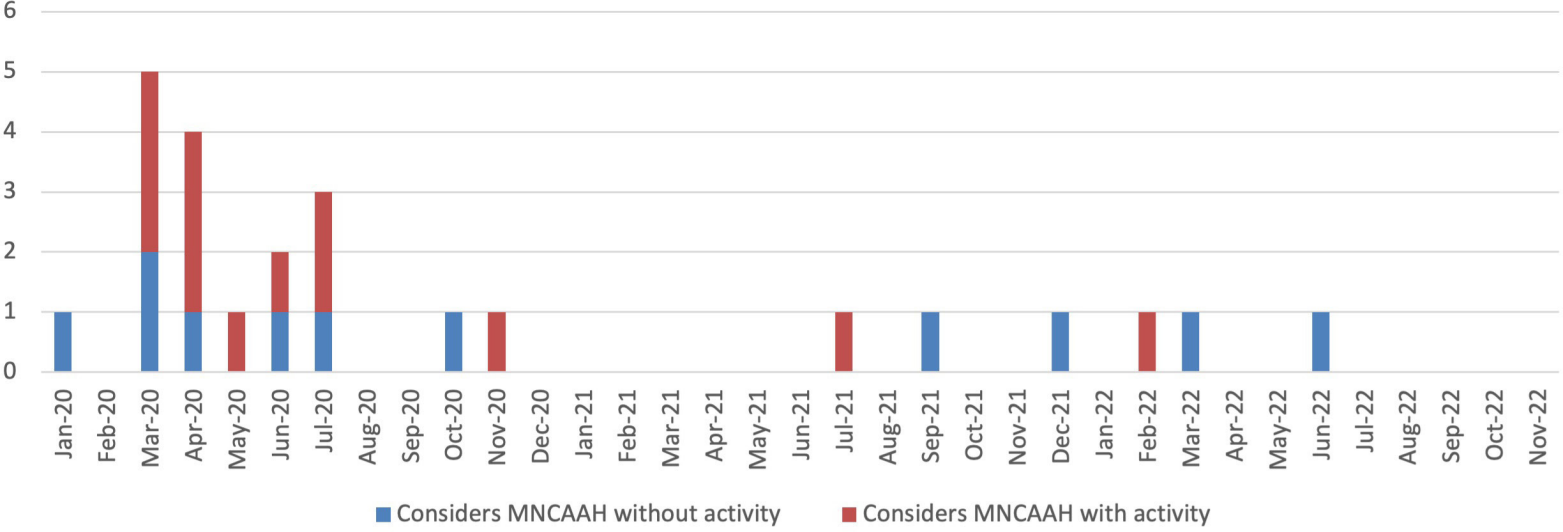
: Publication month of the 24 plans considering the impact of COVID-19 on MNCAAH, with (n=14) and without (n=10) inclusion of an MNCAAH-related activity. MNCAAH, maternal, newborn, child, adolescent and ageing health.

The 24 plans considering the impact of COVID-19 on at least one MNCAAH service were published between January 2020 and February 2022, with 9 (38%) published in March or April (figure 2).

There were 21 plans (19%) published from July 2020 onwards, after the publication of the WHO guidelines setting out clear recommendations for incorporating MNCAAH activities into CPRPs.^25^ Of these, 11 (52%) considered of the impact of the COVID-19 outbreak on one or more MNCAAH service, 5 (24%) included a planned MNCAAH activity, 2 (10%) included indicators for monitoring MNCAAH services or relevant activities, 1 (5%) included costing of activities for an MNCAAH service/s and 1 (5%) detailed the integration of MNCAAH into the wider IMS.

## Discussion

COVID-19 presented countries across the world with the challenge of balancing a direct, rapid outbreak response with the need to maintain essential health service delivery for the whole population. Excess maternal and child mortality as an indirect result of COVID-19 in low-income and middle-income countries was high,^13^ even if it did not reach the levels predicted by early modelling studies.^37 38^ We found that 22% of national CPRPs referenced the impact of COVID-19 on at least one MNCAAH service, but only 13% included a planned activity for monitoring or mitigating this impact and less than 5% included each of: relevant indicators, costing or integration of MNCAAH services in the IMS. No CPRP considered services for all MNCAAH populations. Consideration of services for adolescents and older people was particularly infrequent, as was child health beyond immunisation schedules. This review fills a gap in knowledge about MNCAAH-specific content in publicly available COVID-19 plans, including updates to plans through December 2022.

Early global guidance on MNCAAH service adaption was limited,^24^ but from June 2020 there was a detailed set of WHO-recommended MNCAAH activities and modifications for countries to consider, with recommended indicators to monitor these activities, and recommended ways to incorporate essential services into the IMS.^25^ Around this time, the WHO also began providing intensive support to 19 countries in five WHO Regions to plan and implement actions to mitigate the indirect impacts of COVID-19 on MNCAAH services.^33^ It seems unreasonable to expect that CPRPs—particularly those from low-income and middle-income countries—would have contained interventions, activities and metrics for continuity of health services before the publication of clear WHO guidance to do so, as countries were focused on other core elements of an emergency response emphasised in the initial WHO guidance. A large proportion of the plans found and included in this review were published early in the pandemic: 75% of the CPRPs we identified were published between January and April 2020, and 78% between January and June 2020. The lack of publicly available updates to national plans was also observed by PATH’s COVID-19 Essential Health Services Policy Tracker.^27^ Our findings demonstrate the importance that global guidelines not only integrate essential health service provision from the first publication, but that this integration considers specificities of services including MNCAAH, sexual and reproductive health, nutrition and non-communicable diseases (NCDs). Integrating efforts across health security, disease-specific and life course-specific programmes and objectives and investing in broader health systems capacities to tackle multiple and diverse threats, we can provide greater efficiency and accountability.

Among the 21 plans published or updated after clear WHO guidance was in place (from July 2020 onwards), integration of MNCAAH services was higher yet remained low, for example, 24% of plans published in or after July 2020 included a planned activity for monitoring or mitigating impact on MNCAAH services, versus 10% of those published before July 2020. Because plans were so infrequently updated (at least in a publicly available format), our overall findings are in common with reviews concluded over 2 years before ours. The review of CPRPs up to September 2020 in 106 countries by Mustafa *et al* 26 indicated that 47% of plans considered the impact of COVID-19 on essential health services, and the majority of these plans had limitations in the activities they proposed to mitigate impact on essential services, and/or the integration with the COVID-19 response. Another set of three linked reviews examined CPRPs published up to December 2020 (July 2021 in one review) for evidence relating to the priority setting process.^28–30^ These reviews found common weaknesses in how priorities were set for the use of limited healthcare resources in the context of the pandemic, as judged against a predefined framework for a quality priority setting.

This review found that low-income and middle-income countries were most likely to have a government-authored, published CPRP. Among countries with a published plan, countries classified as low-income, and countries in the South-East Asia region were more likely to have considered the impact on MNCAAH services within the CPRP. This might reflect the tendency for lower-income countries to be more responsive to WHO guidance because of their own current priorities for MNCAAH, the greater need for external support to protect essential services and differences in engagement from WHO national/regional offices. Low- and middle-income countries have greater resource constraints than high-income countries and are therefore likely to develop plans for the purpose of resource mobilisation. Indeed, discussions with Ministries of Health, National Public Health Institutions and planning personnel suggest that developing plans with activity lines and budgets are a key step in approaching external/foreign donors and national funders (eg, finance ministry or funding institutions). In addition, due to existing institutional capacity (eg, strong National Public Health Institutions and/or Centres for Disease Control) and existing laws, regulations, plans and policies on pandemics or emergencies (eg, for influenza, previous SARS or all-hazards emergency/disaster preparedness and response plans, high-income countries may have been adapting and using these instead of developing new plans for COVID-19. High-income countries may also not need to release their plans publicly due to not requiring external/foreign donor resources.

While providing in-depth MNCAAH support to 19 countries, the WHO noted the difficulty that some programmes faced in integrating MNCAAH into COVID-19 response committees and coordination mechanisms, delaying efforts to sustain critical MNCAAH services.^33^ PATH’s COVID-19 Essential Health Services Policy Tracker^27^ similarly found that each health area would often have its own policy development process, some with several documents, and some receiving little attention. This weakened coherence for health workers and sometimes even led to competing guidance. In their review of how health resources were prioritised during the COVID-19 pandemic, Kapiriri *et al* 28 hypothesised that policy makers displayed a ‘knee-jerk’ reaction to reallocate resources to epidemics, following the ‘rule of rescue’—the imperative to respond to the immediate threat to life—even when this is detrimental to the populations most vulnerable to the outbreak. They conclude that there was a true lack of planning for how to rationally allocate resources during the COVID-19 pandemic, despite a sophisticated system and processes for doing so in non-outbreak contexts.

PATH also found evidence of issues with policy implementation, for example while most countries recommended telemedicine to maintain antenatal and postnatal care during COVID-19, only 1% of health workers from low-income countries actually began telemedicine during the pandemic.^27^ In this review, we were limited to exploring the plans and policies stated in published CPRPs, which might have varied considerably from what was implemented. However, although we found some good examples of MNCAAH planning in the CPRPs we reviewed, we observed a lack of operational or implementation detail in general, even among the 14 plans that included an activity relevant to MNCAAH where only four CPRPs included monitoring indicators, and only three included any budget or costing for the activity. It appears that maintenance of essential health services in general, including MNCAAH services, were often not included in the budget and monitoring and evaluation (M&E) sections of CPRPs—an earlier review found that while 88% of CPRPs had a budget component and 53% included an M&E framework, essential services were only reflected in these sections in 24% and 7% of CPRPs, respectively.^26^

The lessons learnt from the COVID-19 pandemic represent an opportunity for future outbreak preparation. Scientists point to a ‘new era’ of infectious disease,^39^ marked by increasing frequency and geographic scope of outbreaks due to the changing climate and land-use patterns, and increased transmission potential aided by increased urbanisation, global travel and trade.^39–41^ The Ebola outbreak in West Africa in 2014–2016 previously showed that mortality and morbidity from indirect effects of an outbreak could match or outweigh the direct impact of the outbreak itself,^42 43^ especially in settings with a high baseline burden of certain health conditions. Policymakers will continue to face difficult decisions when allocating resources between emergency response and routine services, particularly in low resource, fragile or conflict-affected settings. In any setting, the task of maintaining use of essential health services is multifaceted—not just about maintaining supply with the challenges of supply chain disruption, new IPC needs and reduced staffing and resources, but also the need to help people to access and pay for services, address misinformation about services and manage fear of contracting disease while seeking care. This requires systematic and coordinated action to mitigate, and significant contextual adaptation.

The speed and scale of service disruptions seen to essential health services and specifically those for MNCAAH services due to COVID-19^33^ point to significant scope for improvement in ongoing and future planning and policy development. A key learning in this regard is the importance of including personnel from the broader health system (ie, those responsible for essential health service planning, primary healthcare, MNCAAH, etc) in the emergency planning process as well as incident management and response systems. Furthermore, to try and include different stakeholders once a crisis has already started is much more challenging than developing and institutionalising formal mechanisms for collaboration and joint planning during periods of relative normalcy. Therefore, we recommend that routine links are developed, formalised and sustained between those responsible for MNCAAH services and emergency preparedness and response with incentives and accountability mechanisms such as funding, M&E and periodic reporting. This more integrated approach to planning can also be routinely tested using whole-system simulation exercises and delivery of training to key personnel.

Throughout the COVID-19 outbreak, funding for emergency preparedness and response was substantial and subsequent commitments and initiatives are eclipsing what was previously available for example, through the Pandemic Fund, the Independent Panel for Pandemic Preparedness and Response, the global accord on pandemic prevention, preparedness and response. A key consideration for decision makers is to translate the commitments made during the pandemic into building resilient health systems capable of sustainably addressing a broad range of public health needs, including MNCAAH, rather than myopic efforts that focus only on traditional emergency preparedness and response activities. For example, the Pandemic Fund’s initial round of funding focused on three key areas: surveillance and early warning systems, laboratories and the emergency workforce. Alongside this dedicated funding for infectious disease outbreaks, we would advocate for ongoing and future funding efforts and initiatives at global and national level for the consequences of outbreaks. Instead of building up capacities solely for infectious disease surveillance and early warning, we should ensure that systems can detect, prevent and mitigate against a range of potential health hazards, including NCDs (eg, asthma or obesity prevalence in children) and health impacts of essential service disruption (eg, increase in maternal and birth outcomes). Alongside ensuring laboratories have capacity to rapidly test for novel pathogens, they must also be able to detect chemicals and radiation hazards, and conduct genomic sequencing for a broad range of communicable and noncommunicable health threats (eg, common cancers in older people, anaemia in pregnant women or newborn disease screening). As well as strengthening workforce capacities for infectious disease control, efforts should be directed to supporting the workforce to develop broad-based knowledge, competencies and skills, that are transferable and can be deployed for a broad range of public health threats. A more holistic and integrated consideration of population health needs and essential public health functions within policy and planning instruments such as national health sector strategies, public health reforms, all-hazards emergency preparedness and response plans, and national action plans for health security, is crucial to regain and sustain progress towards universal health coverage, health security and improved population and health and well-being.

### Limitations

The main limitation of our study was the lack of publicly available updates to COVID-19 response plans. We did not find a publicly available plan for 5 of the 19 countries that worked intensively with the WHO MNCAAH team, and we only found plans published prior to working with WHO for nine of the other countries. It is possible that MNCAAH planning documents exist separately from the national CPRPs, however we would have expected some of these to be found by our online searches, despite our generic search terms. Where a CPRP linked to or referenced an MNCAAH relevant document, we noted this in our results. Although we included documents in any language, we only searched in English, Spanish, French and Portuguese.

## Conclusions

This review identified very low levels of integration of maternal, newborn, child, adolescent health services, and services for the health of older persons (MNCAAH) into national COVID-19 Response and Recovery plans. The COVID-19 pandemic, along with evidence from earlier outbreaks, showed that mortality and morbidity from indirect effects can match or outweigh the direct impact of the outbreak. The lessons learnt from the COVID-19 pandemic provide an opportunity for future outbreak and emergency preparedness. We recommend that considerations for MNCAAH services are incorporated within future emergency preparedness and response planning including in National Action Plans for Health Security or equivalent. We also recommend that resources and attention from major global resourcing initiatives such as the Pandemic Fund are directed, with appropriate technical support, to strengthen and insure essential service continuation. In the case of future outbreaks and emergencies, we recommend that global guidance includes maintenance of essential services as a key component from the first iteration, with specific guidance for MNCAAH services, and that essential services are integrated into national IMS structures and in national country plans, with accountability mechanisms and access to critical funding and support.

## Data Availability

Data are available upon reasonable request. Data can be obtained from corresponding author.
