# Supplementary material for: National COVID-19 preparedness and response plans: a global review from the perspective of services for maternal, newborn, child and adolescent health and older people
Source: BMJ Glob Health. 2024 Mar 4;9(3):e013711. doi: 10.1136/bmjgh-2023-013711 (PMC10928728; doi:10.1136/bmjgh-2023-013711)
Supplement: Supplementary data [file bmjgh-2023-013711supp001.pdf]

GOOGLE SEARCH TERMS

English
[COUNTRY X] COVID-19 preparedness and response plan
[COUNTRY X] COVID-19 preparedness and response strategy
[COUNTRY X] COVID-19 pandemic plan
[COUNTRY X] COVID-19 national action plan
[COUNTRY X] COVID-19 public health institute

French
[COUNTRY X NAME IN FRENCH] plan de préparation et de riposte COVID-19
[COUNTRY X NAME IN FRENCH] plan de reponse COVID-19
[COUNTRY X NAME IN FRENCH] plan d'action COVID-19
[COUNTRY X NAME IN FRENCH] institut de santé publique COVID-19

Portuguese
[COUNTRY X NAME IN PORTUGUESE] Plano nacional de contingência para o control da epidemia de doença por COVID-19
[COUNTRY X NAME IN PORTUGUESE] Plano de resposta COVID-19
[COUNTRY X NAME IN PORTUGUESE] plano de ação COVID-19
[COUNTRY X NAME IN PORTUGUESE] instituto de saúde pública COVID-19

Spanish
[COUNTRY NAME] Plan de Preparación y Respuesta ante la COVID-19
[COUNTRY NAME] Programa de Emergencia de Apoyo y Preparación ante el COVID-19
[COUNTRY NAME] Plan de acción nacional COVID-19
[COUNTRY NAME] instituto de salud publica COVID-19
